# Supplementary material for: Dynalign II: common secondary structure prediction for RNA homologs with domain insertions
Source: Nucleic Acids Res. 2014 Nov 21;42(22):13939–48. doi: 10.1093/nar/gku1172 (PMC4267632; doi:10.1093/nar/gku1172)
Supplement: SUPPLEMENTARY DATA [file supp_gku1172_nar-02021-z-2014-File012.zip › manual/javadoc/RNAstructure_java_interface/source/windows/package-summary.html]

RNAstructure\_java\_interface.source.windows


JavaScript is disabled on your browser.


- Overview
- Package
- Class
- Tree
- Index
- Help

- Prev Package
- Next Package

- Frames
- No Frames

- All Classes

# Package RNAstructure\_java\_interface.source.windows

A package that holds all internal windows used in the drawing framework.

See: Description

- Class Summary

  | Class | Description |
  | --- | --- |
  |  |  |
  | --- | --- |
  | DrawingWindow | A class responsible for displaying a drawing window. |
  | DynalignFoldWindow | A class responsible for initializing and running the Dynalign module, which calculates common structures for two strands of nucleic acids. |
  | DynalignRefoldWindow | A class responsible for initializing and running the Dynalign refold module, which refolds a previous Dynalign calculation from a save file. |
  | Efn2Window | A class responsible for initializing and running the efn2 (Energy Function 2) module, which calculates the folding free energy of structures. |
  | FoldDoubleWindow | A class responsible for initializing and running the bifold module, which folds two strands of nucleic acids into their likely hybrid structure. |
  | FoldSingleWindow | A class responsible for initializing and running the Fold module, which folds a strand of nucleic acids into their likely lowest free energy structure. |
  | FoldSuboptimalWindow | A class responsible for initializing and running the AllSub module, which calculates the free energy of a varied group of suboptimal structures. |
  | InternalWindow | A class that holds internal frames for the RNAstructure GUI. |
  | MaxExpectWindow | A class responsible for initializing and running the MaxExpect module, which generates a group of structures containing pairs with the maximum probability of being accurate. |
  | ModuleWindow | A class responsible for creating and displaying a window that enables a user to input data for one of the RNAstructure modules. |
  | MultilignWindow | A class responsible for initializing and running the Multilign module, which calculates common secondary structures of multiple sequences using multiple iterations of Dynalign. |
  | MultiWindow | A class responsible for creating and displaying an input window that takes multiple arbitrary sequences and CT files as input. |
  | OligoScreenWindow | A class responsible for initializing and running the OligoScreen module, which calculates thermodynamic properties for a list of oligonucleotides. |
  | OligoWalkWindow | A class that handles creation of windows for the OligoWalk module. |
  | PartitionDoubleWindow | A class responsible for initializing and running the bipartition module, which calculates the partition function for two strands of nucleic acids. |
  | PartitionSingleWindow | A class responsible for initializing and running the partition module, which calculates the partition function for a single strand of nucleic acids. |
  | ProbKnotWindow | A class responsible for initializing and running the ProbKnot module, which predicts pseudoknots in a strand of nucleic acids. |
  | PseudoknotWindow | A class responsible for initializing and running the RemovePseudoknots module, which removes pseudoknots from a nucleic acid sequence. |
  | RefoldWindow | A class responsible for initializing and running the refold module, which refolds a previous folding calculation from a save file. |
  | SequenceDisplayWindow | A class responsible for displaying a window that allows a user to examine raw sequence data. |
  | StochasticWindow | A class responsible for initializing and running the stochastic module, which generates a stochastic sample of structures. |
  | TurboFoldWindow | A class responsible for initializing and running the TurboFold module, which calculates common secondary structures of multiple sequences using the partition function and probabalistic alignments. |


## Package RNAstructure\_java\_interface.source.windows Description

A package that holds all internal windows used in the drawing framework.
  
  
The classes include not only the visual windows, but their actions as well.
These actions include both GUI manipulation and back end calculations.

- Overview
- Package
- Class
- Tree
- Index
- Help

- Prev Package
- Next Package

- Frames
- No Frames

- All Classes
